# 4-(Dimethyl­amino)­pyridinium octa­aqua­erbium(III) tetra­chloride monohydrate

**DOI:** 10.1107/S1600536812043048

**Published:** 2012-10-20

**Authors:** Meriem Benslimane, Hocine Merazig, Jean-Claude Daran, Ouahida Zeghouan

**Affiliations:** a Unité de Recherche de Chimie de l’Environnement et Moléculaire Structurale, Faculté des Sciences Exactes, Département de Chimie, Université Mentouri de Constantine, 25000 Constantine, Algeria; b Laboratoire de Chimie de Coordination, UPR-CNRS 8241, 205 route de Narbonne, 31077 Toulouse Cedex 4, France

## Abstract

In the title compound, (C_7_H_11_N_2_)[Er(H_2_O)_8_]Cl_4_·H_2_O, the asymmetric unit consists of one 4-(dimethyl­amino)­pyridinium and one octa­aqua­erbium cation balanced by four Cl^−^ anions, and one water mol­ecule. The 4-(dimethyl­amino)­pyridinium cation is protonated at the pyridine N atom. The dimethyl­amino group (C/N/C) lies close to the plane of the pyridinium ring, making a dihedral angle of 4.5 (3)°. In the crystal, the [Er(H_2_O)_8_]^3+^ cations are linked *via* O—H⋯O and O—H⋯Cl hydrogen bonds, forming two-dimensional networks propagating in the *ab* plane. These networks are linked *via* O—H⋯O and O—H⋯Cl hydrogen bonds, forming a three-dimensional network. The 4-(dimethyl­amino)­pyridinium cations are located in the cavities and are linked to the framework *via* N—H⋯Cl, C—H⋯O and C—H⋯Cl hydrogen bonds.

## Related literature

For similar structures in this series involving 4-(dimethyl­amino)­pyridinium, see: Benslimane *et al.* (2012*a* ▶,*b* ▶). For details of the Cambridge Structural Database, see: Allen (2002 ▶). For hydrogen-bond motifs see: Bernstein *et al.* (1995 ▶). 
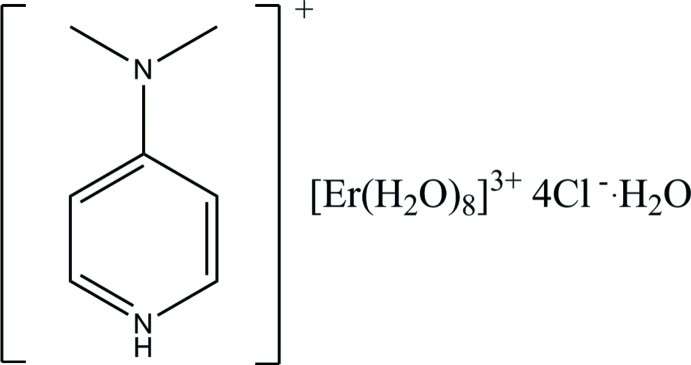


## Experimental

### 

#### Crystal data

(C_7_H_11_N_2_)[Er(H_2_O)_8_]Cl_4_·H_2_O
*M* *_r_* = 594.38
Triclinic, 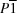

*a* = 7.8775 (3) Å
*b* = 9.3601 (4) Å
*c* = 15.2593 (6) Å
α = 105.831 (3)°
β = 101.498 (3)°
γ = 90.919 (3)°
*V* = 1057.77 (8) Å^3^
*Z* = 2
Mo *K*α radiation
μ = 4.51 mm^−1^
*T* = 180 K
0.35 × 0.17 × 0.09 mm

#### Data collection

Agilent Xcalibur Sapphire1 diffractometer
Absorption correction: multi-scan (*CrysAlis PRO*; Agilent, 2011 ▶) *T*_min_ = 0.415, *T*_max_ = 0.666
21843 measured reflections
4315 independent reflections
4110 reflections with *I* > 2σ(*I*)
*R*_int_ = 0.031

#### Refinement

*R*[*F* ^2^ > 2σ(*F* ^2^)] = 0.015
*wR*(*F* ^2^) = 0.038
*S* = 1.12
4315 reflections
210 parameters
H-atom parameters constrained
Δρ_max_ = 0.38 e Å^−3^
Δρ_min_ = −0.84 e Å^−3^

### 

Data collection: *CrysAlis PRO* (Agilent, 2011 ▶); cell refinement: *CrysAlis PRO*; data reduction: *CrysAlis PRO*; program(s) used to solve structure: *SIR92* (Altomare *et al.*, 1993 ▶); program(s) used to refine structure: *SHELXL97* (Sheldrick, 2008 ▶); molecular graphics: *ORTEPIII* (Burnett & Johnson, 1996 ▶) and *ORTEP-3 for Windows* (Farrugia, 2012 ▶); software used to prepare material for publication: *SHELXL97*.

## Supplementary Material

Crystal structure: contains datablock(s) I, global. DOI: 10.1107/S1600536812043048/su2511sup1.cif

Structure factors: contains datablock(s) I. DOI: 10.1107/S1600536812043048/su2511Isup2.hkl

Additional supplementary materials:  crystallographic information; 3D view; checkCIF report

## Figures and Tables

**Table 1:** Hydrogen-bond geometry (Å, °)

*D*—H⋯*A*	*D*—H	H⋯*A*	*D*⋯*A*	*D*—H⋯*A*
N1—H1⋯Cl1	0.86	2.53	3.229 (2)	139
O1*W*—H1*W*⋯Cl3^i^	0.85	2.44	3.2686 (18)	165
O1*W*—H2*W*⋯Cl3^ii^	0.85	2.25	3.0874 (18)	171
O1—H11⋯Cl4^iii^	0.85	2.29	3.1036 (18)	160
O1—H12⋯Cl1	0.85	2.24	3.0863 (17)	172
O2—H21⋯Cl1	0.85	2.25	3.0708 (17)	164
O2—H22⋯Cl2	0.84	2.31	3.1372 (17)	167
O3—H31⋯O1*W*	0.85	1.82	2.671 (2)	177
O3—H32⋯Cl3	0.84	2.37	3.1826 (17)	162
O4—H41⋯Cl4	0.85	2.25	3.0925 (17)	169
O4—H42⋯Cl2	0.85	2.23	3.0685 (16)	168
O5—H51⋯Cl4	0.85	2.33	3.1469 (18)	160
O5—H52⋯Cl2^iv^	0.85	2.27	3.0819 (18)	161
O6—H61⋯Cl4^v^	0.85	2.27	3.1164 (17)	171
O6—H62⋯Cl1^vi^	0.85	2.25	3.0858 (17)	169
O7—H71⋯Cl3	0.84	2.19	3.0304 (18)	173
O7—H72⋯Cl1^iv^	0.85	2.30	3.1132 (18)	159
O8—H81⋯Cl4^vii^	0.85	2.29	3.1377 (17)	173
O8—H82⋯Cl2^vii^	0.85	2.31	3.1464 (17)	166
C2—H2⋯Cl3^viii^	0.93	2.77	3.683 (3)	169
C3—H3⋯O1*W* ^iii^	0.93	2.51	3.332 (3)	148
C6—H6*B*⋯O4^ii^	0.96	2.47	3.379 (3)	158

Symmetry codes: (i) 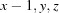; (ii) 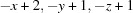; (iii) 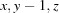; (iv) 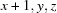; (v) 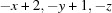; (vi) 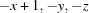; (vii) 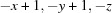; (viii) 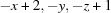.

**Fig. 1.**
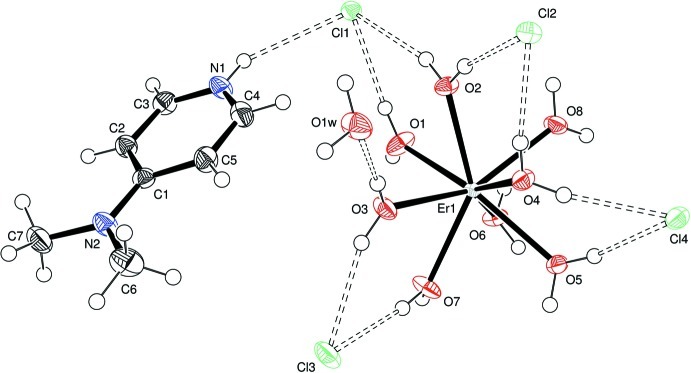
The molecular structure of the title compound, showing the atom-numbering. Displacement ellipsoids are drawn at the 50% probability level. The O-H···Cl and N-H···Cl hydrogen bonds are shown as double dashed lines.

**Fig. 2.**
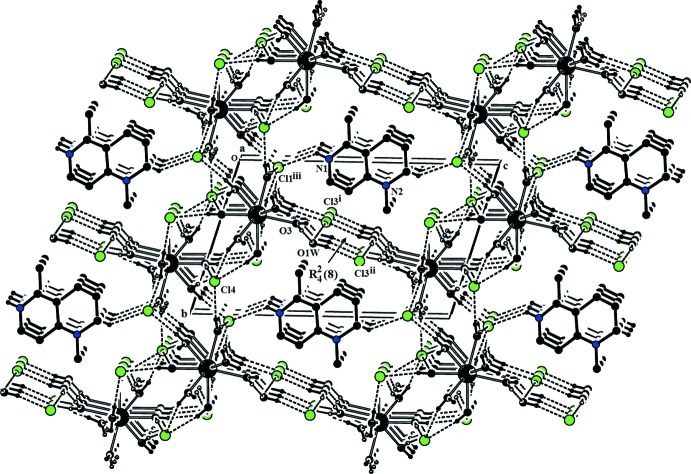
A view of part of the crystal structure of the title compound lying parallel to (100), showing the formation of rings via O-H···Cl and N-H···Cl hydrogen-bonds. Hydrogen bonds are drawn as dashed lines [symmetry codes: (i) x-1, y, z; (ii) -x+2, -y+1, -z+1; (iii) x+1, y, z].

## supplementary crystallographic information

### Comment

The title compound is part of a series of lanthanide complexes with the organic cation 4-(dimethylamino)pyridinium, for example: (C_7_H_10_N_2_)_2_^.^LaCl(H_2_O)_8_^.^Cl_4_^.^3H_2_O (I) (Benslimane *et al.*, 2012*a*) and (C_7_H_10_N_2_)_3_.[Nd_2_Cl_4_(H_2_O)_10_].Cl_5_.2H_2_O (II) (Benslimane *et al.*, 2012*b*).

The title compound (III) contains an inorganic [Er(H_2_O)_8_]^3+^ and an organic (C_7_H_10_N_2_)^+^ cation equilibrated by four Cl anions, and one lattice water molecule (Fig. 1). Atom Er1 is coordinated by eight water molecules with Er-O bond distances ranging from 2.2989 (15) to 2.3807 (15) Å. The [Er(H_2_O)_8_]^3+^ cations are linked to the organic cations via Cl^-^ anions through intermolecular O-H···Cl and N-H···Cl hydrogen bonds. Each Cl^-^ anion acts as an acceptor of hydrogen bonds from the pyridinium groups and the water molecules. The water molecules, which act as bridging units between the cations, form cooperative infinite chains parallel to the (100) plane through O-H···Cl hydrogen bonds generating centrosymmetric R^2^_4_(8) ring motives (Bernstein *et al.*, 1995), as shown in Fig. 2 and Table 1.

In the three compounds, (I) - (III), there is a decrease in the bond lengths of the metal-O(water) bonds, from 2.5101 (15) - 2.5632 (15) Å in (I), 2.404 (3) - 2.479 (4) Å in (II) and 2.2989 (15) - 2.3807 (15) Å in (III). This trend corresponds to the decreasing metallic radius of the lanthanide ion involved; La^3+^, Nd^3+^ and Er^3+^, respectively. In addition, the 4-(dimethylamino)pyridinium cation in the three compounds is protonated at the pyridine N atom. The N-C bond linking the dimethylamino substituent to the pyridinium ring is short, 1.321 (3), 1.324 (3)Å for (I), 1.330 (5), 1.2855 (2) Å for (II) and 1.331 (3) Å for (III), suggesting some delocalization in the cation. A search of the Cambridge Structural Database (CSD, V5.33, Update 4, August 2012; Allen, 2002) reveals similar structures incorporating the 4-(dimethylamino)pyridinium cation for which the corresponding mean N-C distance is 1.34 (1) Å. The dimethylamino group lies close to the plane of the pyridinium ring, with dihedral angles of 3.5 (3) and 2.0 (3)° for (I), 1.6 (6)° and 6.5 (3)° for (II) and 4.5 (3)° for (III).

In conclusion, on the structural level the atomic arrangement in all three compounds, (I) - (III), consists of networks of alternating organic–inorganic layers. The chloride anions are located between these entities forming hydrogen bonds with the NH atoms of the 4-(dimethylamino)pyridinium cations and the water molecules. There are also C—H···Cl interactions present involving one of the 4-(dimethylamino)pyridinium cations. The result is the formation of three-dimensional supramolecular architectures.

### Experimental

4-(Dimethylamino)pyridine (1 mmol, 0.051g) and hydrochloric acid (1M) was added slowly to a solution of ErCl_3_.6H_2_O (1mmol, 0.08g). The mixture was refluxed at 353 K for about 1 h and then cooled to room temperature. Slow evaporation of the solvent at room temperature lead to the formation of pink plate-like crystals of the title compound.

### Refinement

The H-atoms of the coordinated water molecules were located in difference Fourier syntheses and were initially refined using distance restraints: O-H = 0.85 (2) Å, and H···H= 1.40 (2) Å, with *U*_iso_(H) = 1.5*U*_eq_(O). In the last cycles of refinement they were constrained to ride on their parent O atoms. The N-bound H atom was located in a difference Fourier map but like the C-bound H atoms it was included in calculated positions and treated as riding: N-H=0.86 Å, C-H = 0.93 (aromatic), 0.96 (methyl), with *U*_iso_(H) = 1.5*U*_eq_(C) for the methyl groups and 1.2*U*_eq_(N,C) for the other H atoms.

### Crystal data

**Table:**

(C_7_H_11_N_2_)[Er(H_2_O)_8_]Cl_4_·H_2_O	*Z* = 2
*M**_r_* = 594.38	*F*(000) = 586
Triclinic, *P*1	*D*_x_ = 1.866 Mg m^−^^3^
Hall symbol: -P 1	Mo *K*α radiation, λ = 0.71073 Å
*a* = 7.8775 (3) Å	Cell parameters from 17643 reflections
*b* = 9.3601 (4) Å	θ = 2.8–28.5°
*c* = 15.2593 (6) Å	µ = 4.51 mm^−^^1^
α = 105.831 (3)°	*T* = 180 K
β = 101.498 (3)°	Plate, pink
γ = 90.919 (3)°	0.35 × 0.17 × 0.09 mm
*V* = 1057.77 (8) Å^3^	

### Data collection

**Table:**

Agilent Xcalibur Sapphire1 diffractometer	4315 independent reflections
Radiation source: fine-focus sealed tube	4110 reflections with *I* > 2σ(*I*)
Graphite monochromator	*R*_int_ = 0.031
Detector resolution: 8.2632 pixels mm^-1^	θ_max_ = 26.4°, θ_min_ = 2.8°
ω scan	*h* = −9→9
Absorption correction: multi-scan (*CrysAlis PRO*; Agilent, 2011)	*k* = −11→11
*T*_min_ = 0.415, *T*_max_ = 0.666	*l* = −19→19
21843 measured reflections	

### Refinement

**Table:**

Refinement on *F*^2^	Primary atom site location: structure-invariant direct methods
Least-squares matrix: full	Secondary atom site location: difference Fourier map
*R*[*F*^2^ > 2σ(*F*^2^)] = 0.015	Hydrogen site location: inferred from neighbouring sites
*wR*(*F*^2^) = 0.038	H-atom parameters constrained
*S* = 1.12	*w* = 1/[σ^2^(*F*_o_^2^) + (0.0184*P*)^2^ + 0.1863*P*] where *P* = (*F*_o_^2^ + 2*F*_c_^2^)/3
4315 reflections	(Δ/σ)_max_ = 0.013
210 parameters	Δρ_max_ = 0.38 e Å^−^^3^
0 restraints	Δρ_min_ = −0.84 e Å^−^^3^

### Special details

**Table:**

Geometry. All esds (except the esd in the dihedral angle between two l.s. planes) are estimated using the full covariance matrix. The cell esds are taken into account individually in the estimation of esds in distances, angles and torsion angles; correlations between esds in cell parameters are only used when they are defined by crystal symmetry. An approximate (isotropic) treatment of cell esds is used for estimating esds involving l.s. planes.
Refinement. Refinement of F^2^ against ALL reflections. The weighted R-factor wR and goodness of fit S are based on F^2^, conventional R-factors R are based on F, with F set to zero for negative F^2^. The threshold expression of F^2^ > 2sigma(F^2^) is used only for calculating R-factors(gt) etc. and is not relevant to the choice of reflections for refinement. R-factors based on F^2^ are statistically about twice as large as those based on F, and R- factors based on ALL data will be even larger.

### Fractional atomic coordinates and isotropic or equivalent isotropic displacement parameters (Å^2^)

**Table:**

	*x*	*y*	*z*	*U*_iso_*/*U*_eq_	
Er1	0.784214 (11)	0.328931 (10)	0.139815 (6)	0.01301 (4)	
O1	0.6913 (2)	0.08907 (17)	0.13083 (13)	0.0290 (4)	
H11	0.7409	0.0112	0.1094	0.043*	
H12	0.5921	0.0645	0.1382	0.043*	
O2	0.49910 (19)	0.33181 (16)	0.16570 (11)	0.0196 (3)	
H21	0.4352	0.2530	0.1551	0.029*	
H22	0.4382	0.4056	0.1716	0.029*	
O3	0.8148 (2)	0.37949 (19)	0.29989 (11)	0.0246 (4)	
H31	0.7391	0.4245	0.3275	0.037*	
H32	0.9063	0.3747	0.3382	0.037*	
O4	0.71203 (19)	0.58031 (16)	0.18827 (11)	0.0202 (3)	
H41	0.7442	0.6380	0.1593	0.030*	
H42	0.6119	0.6010	0.1983	0.030*	
O5	0.9675 (2)	0.48121 (18)	0.09471 (12)	0.0231 (4)	
H51	0.9316	0.5525	0.0728	0.035*	
H52	1.0724	0.5045	0.1238	0.035*	
O6	0.8996 (2)	0.16376 (17)	0.02461 (11)	0.0222 (3)	
H61	0.9847	0.1931	0.0055	0.033*	
H62	0.8278	0.1097	−0.0222	0.033*	
O7	1.0635 (2)	0.27890 (19)	0.20245 (12)	0.0275 (4)	
H71	1.1090	0.3133	0.2597	0.041*	
H72	1.1158	0.2053	0.1770	0.041*	
O8	0.6080 (2)	0.33727 (19)	0.00199 (11)	0.0253 (4)	
H81	0.4987	0.3160	−0.0103	0.038*	
H82	0.6398	0.3342	−0.0486	0.038*	
N1	0.5425 (3)	−0.0329 (2)	0.34543 (14)	0.0269 (5)	
H1	0.4717	−0.0651	0.2923	0.032*	
N2	0.8825 (3)	0.1213 (2)	0.59717 (14)	0.0255 (4)	
C1	0.7717 (3)	0.0707 (3)	0.51542 (16)	0.0205 (5)	
C2	0.7091 (3)	−0.0813 (3)	0.47840 (17)	0.0228 (5)	
H2	0.7453	−0.1490	0.5117	0.027*	
C3	0.5969 (3)	−0.1280 (3)	0.39497 (17)	0.0250 (5)	
H3	0.5568	−0.2279	0.3715	0.030*	
C4	0.5976 (3)	0.1126 (3)	0.37797 (18)	0.0290 (6)	
H4	0.5576	0.1769	0.3428	0.035*	
C5	0.7091 (3)	0.1666 (3)	0.46032 (18)	0.0264 (5)	
H5	0.7456	0.2674	0.4814	0.032*	
C6	0.9502 (4)	0.2769 (3)	0.6311 (2)	0.0363 (6)	
H6A	0.9889	0.3059	0.5822	0.054*	
H6B	1.0459	0.2891	0.6832	0.054*	
H6C	0.8602	0.3383	0.6504	0.054*	
C7	0.9373 (3)	0.0284 (3)	0.65895 (18)	0.0331 (6)	
H7A	0.8482	−0.0493	0.6484	0.050*	
H7B	0.9571	0.0883	0.7226	0.050*	
H7C	1.0427	−0.0150	0.6465	0.050*	
Cl1	0.32218 (7)	0.03390 (6)	0.16034 (4)	0.01959 (11)	
Cl2	0.33046 (7)	0.63783 (6)	0.19098 (4)	0.02174 (12)	
Cl4	0.78967 (7)	0.76450 (6)	0.05870 (4)	0.02396 (12)	
Cl3	1.20318 (8)	0.38429 (7)	0.41119 (4)	0.03275 (15)	
O1W	0.5751 (2)	0.5134 (2)	0.38849 (12)	0.0326 (4)	
H1W	0.4701	0.4892	0.3866	0.049*	
H2W	0.6335	0.5310	0.4439	0.049*	

### Atomic displacement parameters (Å^2^)

**Table:**

	*U*^11^	*U*^22^	*U*^33^	*U*^12^	*U*^13^	*U*^23^
Er1	0.01068 (5)	0.01438 (6)	0.01302 (6)	0.00048 (4)	0.00106 (4)	0.00335 (4)
O1	0.0269 (9)	0.0144 (8)	0.0502 (12)	0.0028 (7)	0.0212 (8)	0.0073 (8)
O2	0.0153 (8)	0.0132 (7)	0.0304 (9)	0.0007 (6)	0.0061 (7)	0.0052 (7)
O3	0.0201 (8)	0.0383 (10)	0.0147 (8)	0.0082 (7)	0.0020 (7)	0.0075 (7)
O4	0.0182 (8)	0.0181 (8)	0.0250 (9)	0.0000 (6)	0.0059 (7)	0.0062 (7)
O5	0.0150 (8)	0.0236 (9)	0.0343 (10)	0.0008 (6)	0.0043 (7)	0.0149 (8)
O6	0.0164 (8)	0.0263 (9)	0.0194 (8)	−0.0032 (6)	0.0059 (6)	−0.0024 (7)
O7	0.0196 (9)	0.0364 (10)	0.0200 (9)	0.0126 (7)	−0.0023 (7)	0.0014 (7)
O8	0.0152 (8)	0.0432 (11)	0.0168 (8)	0.0004 (7)	0.0002 (6)	0.0097 (8)
N1	0.0245 (11)	0.0337 (12)	0.0179 (10)	−0.0012 (9)	−0.0032 (8)	0.0052 (9)
N2	0.0265 (11)	0.0243 (11)	0.0207 (11)	−0.0009 (8)	−0.0026 (9)	0.0036 (9)
C1	0.0179 (11)	0.0228 (12)	0.0202 (12)	0.0011 (9)	0.0055 (9)	0.0042 (10)
C2	0.0237 (12)	0.0214 (12)	0.0229 (12)	0.0016 (9)	0.0032 (10)	0.0069 (10)
C3	0.0264 (13)	0.0210 (12)	0.0250 (13)	−0.0021 (10)	0.0045 (10)	0.0031 (10)
C4	0.0290 (14)	0.0299 (14)	0.0295 (14)	0.0029 (11)	0.0021 (11)	0.0136 (11)
C5	0.0301 (13)	0.0207 (12)	0.0285 (13)	−0.0004 (10)	0.0032 (11)	0.0091 (10)
C6	0.0373 (15)	0.0268 (14)	0.0338 (15)	−0.0051 (11)	−0.0026 (12)	−0.0021 (12)
C7	0.0323 (14)	0.0390 (16)	0.0246 (14)	0.0000 (12)	−0.0044 (11)	0.0108 (12)
Cl1	0.0179 (3)	0.0183 (3)	0.0215 (3)	−0.0010 (2)	0.0016 (2)	0.0057 (2)
Cl2	0.0182 (3)	0.0212 (3)	0.0242 (3)	0.0015 (2)	0.0031 (2)	0.0048 (2)
Cl4	0.0187 (3)	0.0194 (3)	0.0383 (3)	0.0041 (2)	0.0103 (2)	0.0123 (2)
Cl3	0.0335 (3)	0.0336 (3)	0.0244 (3)	−0.0001 (3)	−0.0101 (3)	0.0088 (3)
O1W	0.0283 (10)	0.0454 (11)	0.0188 (9)	0.0036 (8)	0.0020 (7)	0.0022 (8)

### Geometric parameters (Å, º)

**Table:**

Er1—O8	2.2989 (15)	O8—H82	0.8517
Er1—O1	2.3097 (16)	N1—C3	1.341 (3)
Er1—O3	2.3195 (16)	N1—C4	1.347 (3)
Er1—O7	2.3263 (15)	N1—H1	0.8600
Er1—O5	2.3356 (15)	N2—C1	1.331 (3)
Er1—O6	2.3465 (15)	N2—C6	1.458 (3)
Er1—O2	2.3561 (15)	N2—C7	1.459 (3)
Er1—O4	2.3807 (15)	C1—C2	1.419 (3)
O1—H11	0.8493	C1—C5	1.420 (3)
O1—H12	0.8484	C2—C3	1.352 (3)
O2—H21	0.8455	C2—H2	0.9300
O2—H22	0.8425	C3—H3	0.9300
O3—H31	0.8495	C4—C5	1.344 (4)
O3—H32	0.8439	C4—H4	0.9300
O4—H41	0.8497	C5—H5	0.9300
O4—H42	0.8485	C6—H6A	0.9600
O5—H51	0.8522	C6—H6B	0.9600
O5—H52	0.8499	C6—H6C	0.9600
O6—H61	0.8514	C7—H7A	0.9600
O6—H62	0.8480	C7—H7B	0.9600
O7—H71	0.8439	C7—H7C	0.9600
O7—H72	0.8498	O1W—H1W	0.8471
O8—H81	0.8520	O1W—H2W	0.8491
			
O8—Er1—O1	95.94 (6)	Er1—O6—H61	120.3
O8—Er1—O3	146.14 (6)	Er1—O6—H62	117.0
O1—Er1—O3	86.60 (6)	H61—O6—H62	108.2
O8—Er1—O7	142.03 (6)	Er1—O7—H71	122.0
O1—Er1—O7	88.39 (6)	Er1—O7—H72	124.1
O3—Er1—O7	71.64 (6)	H71—O7—H72	111.0
O8—Er1—O5	81.09 (6)	Er1—O8—H81	122.4
O1—Er1—O5	146.98 (6)	Er1—O8—H82	126.5
O3—Er1—O5	114.04 (6)	H81—O8—H82	108.6
O7—Er1—O5	75.30 (6)	C3—N1—C4	120.7 (2)
O8—Er1—O6	75.79 (6)	C3—N1—H1	119.7
O1—Er1—O6	71.78 (6)	C4—N1—H1	119.7
O3—Er1—O6	135.91 (6)	C1—N2—C6	120.7 (2)
O7—Er1—O6	69.84 (6)	C1—N2—C7	122.8 (2)
O5—Er1—O6	75.65 (6)	C6—N2—C7	116.4 (2)
O8—Er1—O2	74.29 (6)	N2—C1—C2	122.3 (2)
O1—Er1—O2	71.93 (5)	N2—C1—C5	121.6 (2)
O3—Er1—O2	74.51 (6)	C2—C1—C5	116.2 (2)
O7—Er1—O2	141.61 (6)	C3—C2—C1	120.3 (2)
O5—Er1—O2	136.56 (5)	C3—C2—H2	119.9
O6—Er1—O2	129.45 (5)	C1—C2—H2	119.9
O8—Er1—O4	81.90 (6)	N1—C3—C2	121.2 (2)
O1—Er1—O4	141.90 (6)	N1—C3—H3	119.4
O3—Er1—O4	75.79 (6)	C2—C3—H3	119.4
O7—Er1—O4	116.47 (6)	C5—C4—N1	121.3 (2)
O5—Er1—O4	70.61 (5)	C5—C4—H4	119.3
O6—Er1—O4	141.89 (6)	N1—C4—H4	119.3
O2—Er1—O4	70.91 (5)	C4—C5—C1	120.4 (2)
Er1—O1—H11	125.4	C4—C5—H5	119.8
Er1—O1—H12	124.1	C1—C5—H5	119.8
H11—O1—H12	109.5	N2—C6—H6A	109.5
Er1—O2—H21	122.4	N2—C6—H6B	109.5
Er1—O2—H22	125.8	H6A—C6—H6B	109.5
H21—O2—H22	109.7	N2—C6—H6C	109.5
Er1—O3—H31	121.3	H6A—C6—H6C	109.5
Er1—O3—H32	126.0	H6B—C6—H6C	109.5
H31—O3—H32	111.3	N2—C7—H7A	109.5
Er1—O4—H41	115.9	N2—C7—H7B	109.5
Er1—O4—H42	121.1	H7A—C7—H7B	109.5
H41—O4—H42	108.9	N2—C7—H7C	109.5
Er1—O5—H51	122.7	H7A—C7—H7C	109.5
Er1—O5—H52	121.2	H7B—C7—H7C	109.5
H51—O5—H52	108.1	H1W—O1W—H2W	109.7

### Hydrogen-bond geometry (Å, º)

**Table:**

*D*—H···*A*	*D*—H	H···*A*	*D*···*A*	*D*—H···*A*
N1—H1···Cl1	0.86	2.53	3.229 (2)	139
O1*W*—H1*W*···Cl3^i^	0.85	2.44	3.2686 (18)	165
O1*W*—H2*W*···Cl3^ii^	0.85	2.25	3.0874 (18)	171
O1—H11···Cl4^iii^	0.85	2.29	3.1036 (18)	160
O1—H12···Cl1	0.85	2.24	3.0863 (17)	172
O2—H21···Cl1	0.85	2.25	3.0708 (17)	164
O2—H22···Cl2	0.84	2.31	3.1372 (17)	167
O3—H31···O1*W*	0.85	1.82	2.671 (2)	177
O3—H32···Cl3	0.84	2.37	3.1826 (17)	162
O4—H41···Cl4	0.85	2.25	3.0925 (17)	169
O4—H42···Cl2	0.85	2.23	3.0685 (16)	168
O5—H51···Cl4	0.85	2.33	3.1469 (18)	160
O5—H52···Cl2^iv^	0.85	2.27	3.0819 (18)	161
O6—H61···Cl4^v^	0.85	2.27	3.1164 (17)	171
O6—H62···Cl1^vi^	0.85	2.25	3.0858 (17)	169
O7—H71···Cl3	0.84	2.19	3.0304 (18)	173
O7—H72···Cl1^iv^	0.85	2.30	3.1132 (18)	159
O8—H81···Cl4^vii^	0.85	2.29	3.1377 (17)	173
O8—H82···Cl2^vii^	0.85	2.31	3.1464 (17)	166
C2—H2···Cl3^viii^	0.93	2.77	3.683 (3)	169
C3—H3···O1*W*^iii^	0.93	2.51	3.332 (3)	148
C6—H6*B*···O4^ii^	0.96	2.47	3.379 (3)	158

Symmetry codes: (i) *x*−1, *y*, *z*; (ii) −*x*+2, −*y*+1, −*z*+1; (iii) *x*, *y*−1, *z*; (iv) *x*+1, *y*, *z*; (v) −*x*+2, −*y*+1, −*z*; (vi) −*x*+1, −*y*, −*z*; (vii) −*x*+1, −*y*+1, −*z*; (viii) −*x*+2, −*y*, −*z*+1.
